# Melanopsin‐Mediated Post‐Illumination Pupillary Response in Idiopathic Rapid Eye Movement (REM) Sleep Behavior Disorder and Parkinson's Disease

**DOI:** 10.1002/mds.70412

**Published:** 2026-06-29

**Authors:** Joey W.Y. Chan, Bei Huang, Siyi Gong, Yuhua Yang, Shi Tang, Steven Wai‐ho Chau, Ngan Yin Chan, Tim Man‐ho Li, Yaping Liu, Anne Y.Y. Chan, Karen K.Y. Ma, Shirley X. Li, Joshua Tsoh, Kelvin K.L. Chong, Kathryn A. Roecklein, Vincent C.T. Mok, Yun Kwok Wing

**Affiliations:** ^1^ Li Chiu Kong Family Sleep Assessment Unit, Department of Psychiatry, Faculty of Medicine The Chinese University of Hong Kong Hong Kong China; ^2^ Center for Sleep and Circadian Medicine The Affiliated Brain Hospital of Guangzhou Medical University Guangzhou China; ^3^ Key Laboratory of Neurogenetics and Channelopathies of Guangdong Province and the Ministry of Education of China Guangzhou Medical University Guangzhou China; ^4^ Department of Medicine and Therapeutics, Faculty of Medicine The Chinese University of Hong Kong Hong Kong China; ^5^ Department of Psychology The University of Hong Kong Hong Kong China; ^6^ Department of Psychiatry Prince of Wales Hospital Hong Kong China; ^7^ Department of Ophthalmology and Visual Science The Chinese University of Hong Kong Hong Kong China; ^8^ Department of Psychology University of Pittsburgh Pittsburgh Pennsylvania USA; ^9^ Li Ka Shing Institute of Health Sciences, Faculty of Medicine The Chinese University of Hong Kong Hong Kong China; ^10^ Gerald Choa Neuroscience Institute The Chinese University of Hong Kong Hong Kong China

**Keywords:** circadian rhythms, post‐illumination pupillary response, Parkinson's disease, REM sleep behavior disorder

## Abstract

**Aims:**

To conduct a case–control study to investigate melanopsin‐mediated post‐illumination pupillary response (PIPR) in patients with Parkinson's disease (PD), video‐polysomnography‐confirmed isolated/idiopathic rapid eye movement (REM) sleep behavior disorder (iRBD), and age‐matched healthy controls (HC).

**Methods:**

PIPR was measured at 6 s after light offset (PIPR‐6s). Net PIPR‐6s was calculated by subtracting the red‐light response from the blue‐light response. Participants also underwent 1‐week actigraphy, overnight urinary 6‐sulfatoxymelatonin assessment, and cognitive testing using the Hong Kong Montreal Cognitive Assessment (HK‐MoCA).

**Results:**

We recruited 135 participants (mean age 64.4 ± 5.7 years; 56% male), with 45 in each group. Net PIPR‐6s was 23.8 ± 9.4% in HC, 18.6 ± 10.8% in iRBD, and 13.3 ± 9.6% in PD (*P* < 0.001; HC > iRBD > PD). Net PIPR‐6s was positively associated with circadian rest–activity rhythm amplitude, mesor, and HK‐MoCA score.

**Conclusion:**

Attenuated PIPR may reflect early dysfunction in melanopsin‐mediated phototransduction in the prodromal stage of synucleinopathy. © 2026 The Author(s). *Movement Disorders* published by Wiley Periodicals LLC on behalf of International Parkinson and Movement Disorder Society.

Parkinson's disease (PD) is an alpha‐synucleinopathy characterized by significant degeneration of nigrostriatal dopaminergic neurons and other areas leading to motor and non‐motor symptoms.^1^ Emerging evidence shows that circadian rhythm disturbances are closely associated with PD.^2^, ^3^ The retinal changes that are associated with dysfunctional melanopsin‐mediated responses may explain circadian disruptions in PD.^4^ Melanopsin‐containing intrinsically photosensitive retinal ganglion cells (ipRGCs) project to the suprachiasmatic nucleus (SCN) to mediate melatonin secretion and circadian entrainment,^5^ and to the midbrain to mediate pupillary response.^6^ By using blue light stimuli of 480 nm that preferentially stimulates melanopsin‐ipRGCs, the continuing constriction of the pupil after light‐off (ie, the post‐illumination pupillary response [PIPR]) represents a proxy marker of the intrinsic melanopsin‐ipRGC function.^7^

Attenuation of PIPR has been demonstrated in patients with PD,^8^ but data in idiopathic/isolated rapid eye movement (REM) sleep behavior disorder (iRBD) is more limited. iRBD is a strong prodromal marker of alpha‐synucleinopathies, with over 80% of the iRBD patients progressing to PD or related neurodegenerative disorders in 14 years.^9^, ^10^ A case–control study of 16 iRBD patients found no difference in melanopsin‐mediated PIPR from the control group^11^; but in a larger series of 69 patients with iRBD, PIPR was significantly correlated with cognitive domains and was also more attenuated in patients with mild cognitive impairment than those without, albeit that there was no control group for comparison.^12^ Our prior work demonstrated that an attenuated PIPR is linked to age‐related circadian alterations, including reduced rest–activity rhythm (RAR) amplitude and mesor.^13^ This study extends previous findings by comparing melanopsin‐mediated PIPR across healthy controls (HC), iRBD, and PD subjects. We hypothesized that PIPR would be progressively attenuated across controls, iRBD, and PD patients, which would be associated with a dampened RAR amplitude and mesor and lower cognitive scores.

## Methods

### Study Design and Subject Recruitment

This was a case–control study conducted in a university‐affiliated sleep clinic. Diagnosis of PD was made by neurologists. All patients with iRBD were confirmed by video‐polysomnography. Controls were age‐matched individuals with no history of iRBD, psychiatric disorders, or neurodegenerative diseases. The inclusion and exclusion criteria are described in Supplement S1. This study adheres to the Strengthening the Reporting of Observational Studies in Epidemiology (STROBE) Statement for reporting.^14^

### Measurements

Each participant completed: (i) REM Sleep Behavior Disorder Questionnaire‐Hong Kong (RBDQ‐HK),^15^ (ii) Insomnia Severity Index (ISI),^16^ and (iii) The Patient Health Questionnaire‐9 (PHQ‐9).^17^ Motor symptoms in patients with PD were evaluated by means of the Unified Parkinson's Disease Rating Scale‐Part III (UPDRS‐III).^18^ A trained research assistant conducted the HK‐MoCA.

### Actigraphy

All participants were asked to wear an actiwatch (Actiwatch Spectrum Plus, Respironics, Philips) on the non‐dominant wrist for 1 week. Actigraphically assessed RAR were examined by the nonparametric circadian rhythm analysis (NPCRA)^19^ and cosinor analysis.^20^

### Urinary 6‐Sulfatoxymelatonin

Subjects were advised to empty their bladder before bed and collect all the urine outputs from 22:00 until the morning void on the next day. All 6‐sulfatoxymelatonin (aMT6s) levels were creatinine‐adjusted (details in Supplement S2).

### Pupillometric Measurements

The PIPR was measured with the NeurOptics DP‐2000 Pupillometer (Irvine, CA, USA). PIPR‐6s measured the pupil size at 6 s post‐stimulus divided by the baseline pupil diameter (BPD).^21^ The Net PIPR‐6s were reported by subtracting the PIPR‐red from the PIPR‐blue and was the predefined outcome in this study (Supplement S3).

### Statistical Analysis

Ordinal logistic regression assessed the associations between PIPR and disease status (HC < iRBD < PD). Generalized linear models examined the relationships between PIPR and actigraphic amplitude, mesor, acrophase, and urinary aMT6s. Linear regressions tested associations with HK‐MoCA scores, adjusted for confounders. Sensitivity analyses were conducted excluding three iRBD participants taking antidepressants, and additional analysis excluding 18 participants using melatonin were conducted for sleep and circadian outcomes. Statistical tests were performed using SPSS (v27.0) for Windows (IBM Corp.) and R Studio (Version 4.5.1). (Supplement S4).

## Results

### Demographic and Clinical Characteristics

A total of 45 healthy controls, 45 patients with iRBD, and 45 patients with PD were recruited (mean ± SD: 64.6 ± 5.7 years) (Table 1). There was a difference in the percentage of males (*P* = 0.02). The PD group had the highest ISI and PHQ‐9 scores, compared with iRBD and HC, respectively. None of the HC met the RBDQ‐HK cut off (18/19) for iRBD.^15^ Benzodiazepines were prescribed to 56% of PD patients and 40% of iRBD patients, while 38% of the iRBD group received melatonin. Forty‐two (93%) PD patients were receiving dopaminergic medications.

**Table 1:** Basic demographic and clinical characteristics.

Characteristic	All	HC^1^	iRBD^2^	PD^3^	*P*‐value	Post‐hoc test
	(N = 135)	(N = 45)	(N = 45)	(N = 45)		
Demographics, mean ± SD						
Age, years	64.6 ± 5.7	63.2 ± 5.7	64.8 ± 4.5	65.7 ± 6.5	0.12	NA
Education, years	11.1 ± 5.1	11.6 ± 4.8	11.2 ± 6.2	10.4 ± 4.1	0.50	NA
Male, n (%)	75 (56)	19 (42)	32 (71)	24 (53)	0.02^g^	NA
HK‐MoCA	26.7 ± 2.6	27.4 ± 2.1	26.5 ± 2.6	26.3 ± 2.9	0.11	NA
ISI	8.7 ± 7.1	8.0 ± 5.8	7.1 ± 4.8	11.1 ± 9.3	0.02	2 < 3
PHQ‐9	5.5 ± 8.2	2.8 ± 3.4	5.3 ± 12.2	8.6 ± 5.1	0.003	1 < 3
RBDQ‐HK Total	24.1 ± 20.1	5.5 ± 4.8	36.0 ± 16.2	30.9 ± 19.8	<0.001	1 < 2, 1 < 3
RBDQ‐HK Total >18, n (%)	68 (50)	0 (0)	37 (82)	31 (69)	<0.001	1 < 2
Duration of PD, years	NA	NA	NA	10.1 ± 5.3	NA	NA
UPDRS‐III	NA	NA	NA	24.1 ± 10.4	NA	NA
HY‐Staging	NA	NA	NA	2.0 ± 0.7	NA	NA
Medications, n (%)						
Antihypertensives^a^	29 (21)	8 (18)	9 (20)	12 (27)	0.57	NA
Anti‐DM medications^b^	12 (9)	3 (7)	3 (7)	6 (13)	0.44	NA
Anti‐lipid drugs^c^	27 (20)	9 (20)	10 (22)	8 (18)	0.87	NA
Anti‐depressants^d^	7 (5)	0 (0)	3 (7)	4 (9)	0.14	NA
Benzodiazepines^e^	43 (32)	0 (0)	18 (40)	25 (56)	<0.001	1 < 2 < 3
Exogenous melatonin	18 (13)	0 (0)	17 (38)	1 (2)	<0.001	1 <3 < 2
PD medications^f^	0 (0)	0 (0)	0 (0)	42 (93)	NA	NA
LEDD	NA	NA	NA	612.9 ± 310.0	NA	NA

*Note:* The superscript numbers 1–3 here represent the respective group for the column post‐hoc test.

Abbreviations: DM, diabetes mellitus; HC, healthy controls; HK‐MoCA, Hong Kong‐Montreal Cognitive Assessment; HY, Hoehn and Yahr; iRBD, idiopathic rapid eye movement (REM) sleep behavior disorder; ISI, Insomnia Severity Index; LEDD: levodopa equivalent daily dose; NA, not applicable; PD, Parkinson's disease; PHQ‐9, Patient Health Questionnaire‐9; RBDQ‐HK, REM Sleep Behavior Disorder Questionnaire‐Hong Kong; SD, standard deviation; UPDRS‐III, Unified Parkinson's Disease Rating Scale‐Part III.

^a^ Antihypertensives included linsinopril, amlodipine, and losartan.

^b^ Anti‐DM medications included metformin and alogliptin.

^c^ Anti‐lipid medications included simvastatin and atorvastatin.

^d^ Antidepressants included paroxetine and sertraline.

^e^ Benzodiazepines included clonazepam, diazepam, and alprazolam.

^f^ PD medications included rasagiline, ropinirole, pramipexole, madopar, rotigotine, sinemet, amantadine, and seligiline.

^g^ The standardized residuals show that no single group (HC, iRBD, or PD) has a distribution that differs significantly from the expected distribution.

### Actigraphic Rest–Activity Measures and aMT6s

The PD group had higher wakefulness after sleep onset (WASO) and a lower sleep efficiency than the control group. Additionally, PD patients had a lower interdaily stability (IS), higher intradaily variability (IV), lower average activity during the most active 10‐hour period (M10), and lower mesor and amplitude, when compared with both HC and iRBD. The overnight urinary aMT6s levels did not differ among the three groups (Supplementary Table S1). Additional sensitivity analysis by excluding the subjects on melatonin did not alter the main findings (Table S2).

### Pupillometric Measurements

The Net PIPR‐6s were progressively attenuated across the HC, iRBD, and PD groups (23.8 ± 9.4% vs. 18.6 ± 10.8% vs. 13.3 ± 9.6%, *P* < 0.001, post‐hoc: HC>iRBD>PD) (Fig. 1). PIPR‐6s to blue stimulus were lower in the PD group compared with the HC group (34.0 ± 10.6% vs. 22.8 ± 11.4%, *P* < 0.001), whereas no differences were observed in PIPR‐6s to red stimuli and the baseline pupil diameter (Table S3). The progressive attenuation remained significant after excluding three iRBD patients who were taking antidepressant medication (23.8 ± 9.4% vs. 19.2 ± 10.5% vs. 13.3 ± 10.7%, *P* < 0.001).

**FIG. 1:**
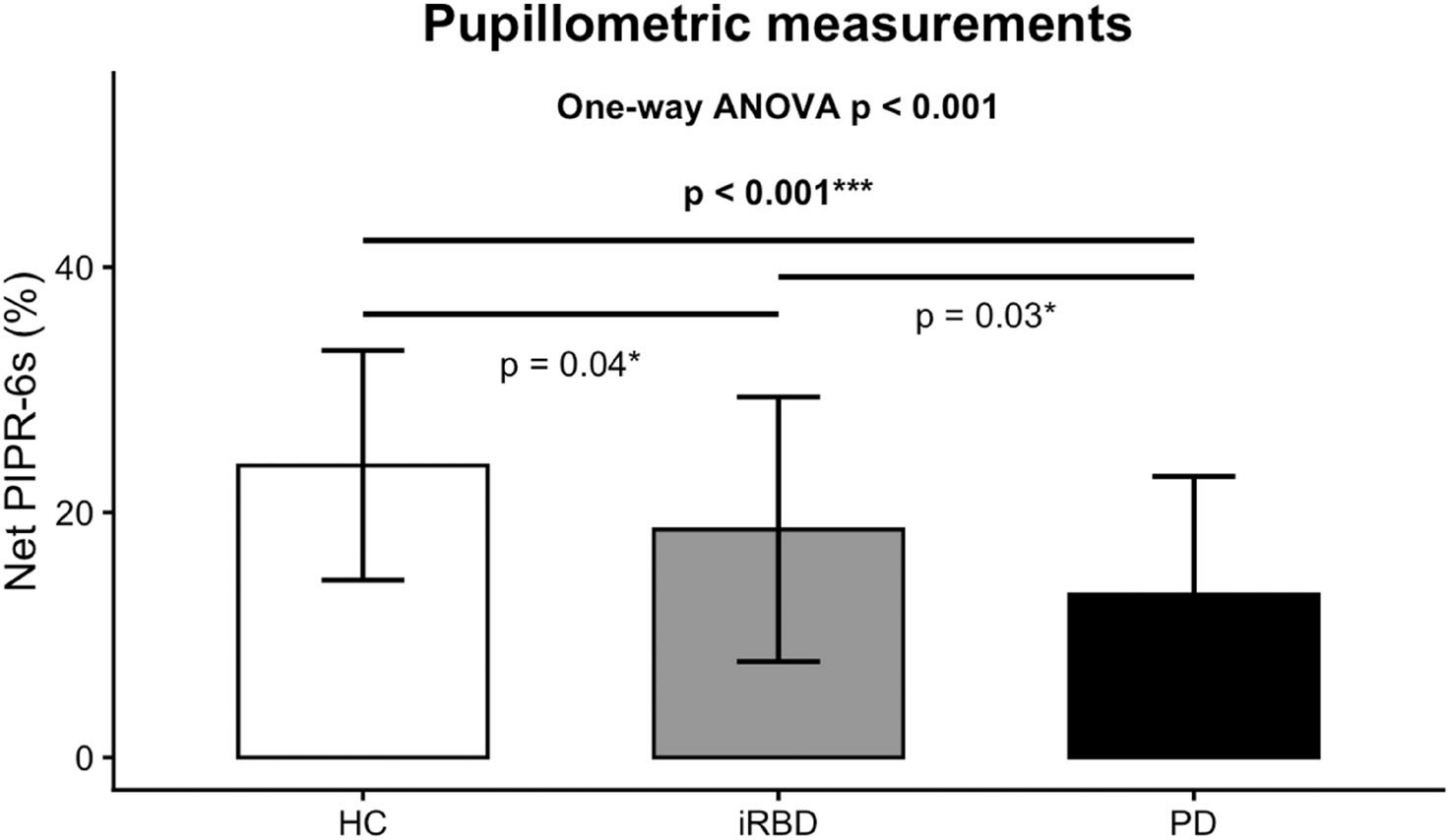
Net post‐illumination pupillary response measured at 6 s after light offset (PIPR‐6s) among the three groups. HC, healthy controls; iRBD, idiopathic rapid eye movement (REM) sleep behavior disorder; PD, Parkinson's disease.

### Association of PIPR with the Risk of iRBD and PD

The ordinal logistic regression of the three groups (HC, iRBD, PD) represented ordered progression of the neurodegenerative spectrum. A stronger Net PIPR‐6s was independently associated with a reduced risk (odds ratio [OR] 0.92; 95% CI: 0.88, 0.95; *P* < 0.001) of being in the categories of the iRBD and PD groups. The findings persisted in the sensitivity analyses (Table S4).

### Association of PIPR with Reduced Circadian Amplitude and Mesor, and Cognitive Functions

Generalized linear models (GLMs) showed that Net PIPR‐6s was negatively associated with circadian RAR amplitude and mesor (Table S5). Linear regression models found significant positive association between Net PIPR‐6s and HK‐MoCA, adjusting for age, gender, education, ISI, and PHQ‐9 scores (Table S6).

## Discussion

Our study demonstrated a progressive attenuation of the melanopsin‐mediated PIPR across the alpha‐synucleinopathy spectrum. It extended previous studies of attenuated PIPR among patients with PD^4^, ^8^, ^22^ and found that the attenuation occurred early in RBD patients compared with controls. Furthermore, Net PIPR‐6s was significantly associated with circadian mesor, amplitude, and HK‐MoCA score across the groups.

### Changes in Sleep and Circadian Rhythms in PD and iRBD

The actigraphic measurement found higher WASO and lower sleep efficiency in PD patients, which corroborated with other studies that reported an increase in self‐reported nocturnal awakenings, disturbed sleep maintenance, and increased WASO in PD patients.^23^, ^24^, ^25^ Consistent with prior studies, PD patients in our study showed lower interdaily stability (IS), higher intradaily variability (IV), lower RAR mesor, and amplitude.^3^, ^26^ However, iRBD did not differ significantly from controls on most cosinor or nonparametric RAR measures. A preliminary case–control study also showed no differences in IS and IV in the iRBD group (N = 18), but lower M10, relative amplitude, and total salivary melatonin level were observed.^27^ This aligns with our previous work, which demonstrated an increase in activity fragmentation, and physical inactivity during active periods in patients with iRBD compared with controls.^28^ Collectively, the current evidence suggests pronounced circadian RAR changes in PD, which may begin to emerge in iRBD.

### Retinal Phototransduction as a Mechanism of Circadian Disruptions in PD and iRBD

The melanopsin‐containing ipRGCs, which are the conduit for conveying light signals to regulate circadian rhythms, have marked reduction in cell density and number of intersections in post‐mortem examination in PD patients.^29^, ^30^ The level of alpha‐synuclein deposits in the retina was correlated with the severity of PD^31^ and suggested that the impairment of the retinal melanopsin system may represent a biomarker of neurodegeneration in PD.^32^ Our previous work found an association of attenuated PIPR with lower circadian amplitude and mesor among older healthy individuals,^13^ and we replicated this finding in this study among subjects across various stages of alpha‐synucleinopathies. Collectively, these findings suggest that degenerative changes in PD may have accentuated the normal aging process of the retinal‐ipRGC‐circadian system.

### PIPR and Cognitive Functions

Net PIPRs were significantly associated with HK‐MoCA scores in our study. The results are consistent with the study by Steiner et al. who reported that PIPR correlated with executive functions among iRBD patients, and patients with mild neurocognitive disorder (N = 10) had significantly reduced PIPR to blue stimulus compared with those without.^12^

### Clinical Implications and Future Research Direction

Our study demonstrated that Net PIPR‐6s was significantly attenuated in both iRBD and PD patients, which was associated with reduced circadian and cognitive function. While promising as a marker of disease severity, prospective longitudinal studies of PIPR will be needed to determine its utility in predicting phenoconversion and monitoring of impaired cognition of iRBD. In addition, as the effects of bright light therapy in regulating the circadian dysfunction and sleep among patients with PD is mixed,^33^ further research should investigate whether PIPR may serve as a convenient and clinically accessible biomarker in predicting the effectiveness of light interventions.

## Limitations

First, formal ophthalmological examination, such as slit‐lamp examination or quantification of lens opacity, was not done in the present study. Additionally, abnormal pupillary dynamics can be related to autonomic dysfunction.^11^, ^34^ In our study, no significant differences were observed in PIPR‐6s to red stimuli and baseline pupil diameter among the three groups. The selective reduction in blue/Net‐PIPR without corresponding changes in red‐light responses or baseline pupil size suggests that generalized autonomic impairment alone may not fully account for the observed melanopsin‐related PIPR abnormality. Nevertheless, autonomic function measures are required in future studies to disentangle the effects between autonomic system and melanopsin‐mediated phototransduction. Second, not all the PD patients had a preceding polysomnography‐confirmed diagnosis of iRBD, and caution is required when generalizing our findings to other alpha‐synucleinopathies. Third, benzodiazepine use was common and may have influenced sleep–wake patterns, although benzodiazepines did not influence pupillary response despite their sedative property.^21^ The high percentage of patients on benzodiazepine likely reflects the treatment need for iRBD symptoms and other sleep disturbances,^35^ which make our sample more generalizable to patients in clinical practice. Lastly, dopaminergic drugs may influence circadian rhythm,^36^ and future studies should consider recruitment of drug‐naive patients.

## Author Roles

(1) Research Project: A. Conception, B. Organization, C. Execution; (2) Statistical Analysis: A. Design, B. Execution, C. Review and Critique; (3) Manuscript Preparation: A. Writing of the First Draft, B. Review and Critique; (4) Other: A. Funding Acquisition.

J.W.Y.C.: 1A, 1B, 1C, 2A, 2B, 2C, 3A, 3B, 4A.

B.H.: 1C, 2C, 3B.

S.G.: 1C, 3B.

Y.Y.: 1C, 3B.

S.T.: 1C, 3B.

S.W.C.: 1A, 1C, 3B.

N.Y.C: 3B.

T.M.L.: 3B.

Y.L.: 1C, 3B.

A.Y.Y.C.: 1C, 3B.

K.K.Y.M.: 1C, 3B.

S.X.L.: 3B.

J.T.: 3B.

K.K.L.C.: 3B.

K.A.R.: 1A, 3B.

V.C.T.M.: 1A, 1C, 3B.

Y.K.W.: 1A, 1B, 1C, 2C, 3A, 3B, 4A.

## Financial Disclosures for the Previous 12 Months

J.W.Y.C. received grants from Takeda Inc., and travel support from Lundbeck HK Ltd for overseas conferences. K.K.L.C. received consulting fees from Amgen Inc. Y.K.W. received grants from Takeda Inc., and sponsorship from Lundbeck HK Ltd and Aculys Pharma, Inc. The remaining authors declare that the study was conducted in the absence of any commercial or financial relationships that could be construed as a potential conflict of interest.

## Financial Disclosures and Conflicts of Interest

Author disclosures are available in the Supporting Information.

## Supporting information


**Supplementary S1.** Inclusion and exclusion criteria.
**Supplementary S2**. Collection of overnight 6‐sulfatoxymelatonin (aMT6s).
**Supplementary S3**. Pupillometric measurements.
**Supplementary S4**. Statistical analysis.
**Table S1.** Actigraphic rest–activity rhythms and melatonin.
**Table S2.** Sensitivity analysis excluding subjects on exogenous melatonin.
**Table S3.** Pupillometric measurements.
**Table S4.** Ordinal logistic regression analysis.
**Table S5.** Generalized linear model of net post‐illumination pupillary response (PIPR) on circadian parameters.
**Table S6.** Linear regression analysis of net post‐illumination pupillary responses (PIPRs) with Hong Kong Montreal Cognitive Assessment (HK‐MoCA) score.

COI_Disclosure

## Data Availability

Deidentified data from this study can be shared upon reasonable request to the corresponding author by a qualified investigator whose proposed use of the data has been approved.
