# Supplementary material for: Melanopsin‐Mediated Post‐Illumination Pupillary Response in Idiopathic Rapid Eye Movement (REM) Sleep Behavior Disorder and Parkinson's Disease
Source: Mov Disord. 2026 Jun 29;41(10):2706–11. doi: 10.1002/mds.70412 (PMC13686108; doi:10.1002/mds.70412)
Supplement: Supplementary file 2 [file MDS-41-2706-s001.docx]

Supplementary material

**Melanopsin-mediated post-illumination pupillary response in idiopathic REM sleep behavior disorder and Parkinson’s disease**

Joey WY Chan, MBChB,^1^* Bei Huang, PhD,^1^ Siyi Gong, PhD, ^1^ Yuhua Yang, PhD, ^1^ Shi Tang, PhD, ^1^ Steven Wai-ho Chau, MBChB,^1^ Ngan Yin Chan, PhD,^1^ Tim Man-ho Li, PhD,^1^ Yaping Liu, MD, PhD,^2,3^ Anne YY Chan, MD,^4^ Karen KY Ma, MD,^4^ Shirley X Li, PhD,^5^ Joshua Tsoh, MBChB,^6^ Kelvin KL Chong, MBChB,^7^ Kathryn A Roecklein, PhD,^8^ Vincent CT Mok, MD,^4^ Yun Kwok Wing, MBChB^1,9^

Table of Contents

[Supplement 1. Inclusion and exclusion criteria	2](#_Toc231311780)

[Supplement 2. Collection of overnight aMT6s	3](#_Toc231311781)

[Supplement 3. Pupillometric measurements	4](#_Toc231311782)

[Supplement 4. Statistical analysis	5](#_Toc231311783)

[eTable 1: Actigraphic rest-activity rhythms and melatonin	6](#_Toc231311784)

[eTable 2. Sensitivity analysis excluding subjects on exogenous melatonin	7](#_Toc231311785)

[eTable 3. Pupillometric measurements	8](#_Toc231311786)

[eTable 4. Ordinal logistic regression analysis	9](#_Toc231311787)

[eTable 5. Generalized linear model of Net PIPR on circadian parameters	10](#_Toc231311788)

[eTable 6. Linear regression analysis of Net PIPRs with HK-MoCA score	11](#_Toc231311789)

[Reference	13](#_Toc231311790)

## Supplement 1. Inclusion and exclusion criteria

Patients with PD were recruited from an on-going trial of bright light therapy for PD patients (ClinicalTrials.gov ID: NCT05524961). All patients with PD in this study had a clinical diagnosis of PD confirmed by neurologists according to the standard diagnostic criteria of PD^1^. Only baseline data collected prior to any intervention were being analysed in the present study. Patients with iRBD were referred from their treating clinicians from the sleep clinic. iRBD was diagnosed according to the International Classification of Sleep Disorder – third edition (ICSD-3-TR)^2^ which has been confirmed by video-polysomnography, and the iRBD symptoms were not secondary to other sleep or neurological diseases (e.g., narcolepsy and severe sleep apnea). iRBD patients who were diagnosed with any types of neurodegenerative diseases were excluded. Healthy controls were recruited from the community through email, flyers and social media. They were age-matched controls with no prior history of iRBD, psychiatric disorders or neurodegenerative diseases. The following exclusion criteria applied to all participants: i) use of oral medication (e.g. anti-cholinergic, beta-blockers or sympathomimetic drugs), or topical eye drops that affects pupillary response, ii) known history of eye surgery, intraocular lens, pupil irregularity, retinal eye disease, severe cataract, glaucoma, color blindness, and blindness, iii) significant medical condition/ hearing impairment/ speech deficit leading to incapability of completing clinical interview, iv) regular shift-workers or trans-meridian flight in the past three months, v) a Hong Kong version of Montreal Cognitive Assessment (HK-MoCA) score below 22, which is a validated cut-off to differentiate cognitive impaired persons (mild cognitive impairment and dementia) from normal controls^3^, vi) inability to give written informed consent.

## Supplement 2. Collection of overnight aMT6s

An overnight urine sample was collected from all participants except for the following conditions: i) those who are on exogenous melatonin, oral contraceptive pills, beta-blocker, NSAIDs which were known to decrease nocturnal melatonin production^4,5^ and ii) patients with unstable medical condition, renal or hepatic impairments. Melatonin is metabolized mainly in the liver to 6-hydroxymelatonin and then conjugated with sulfuric acid to 6-sulfatoxymelatonin, which is the main metabolites excreted in the urine ^6^. It has been demonstrated that the morning aMT6 correlates well with nocturnal melatonin secretion ^7^ and could serve as a non-invasive technique for monitoring melatonin. Subjects were advised to abstain from caffeine, banana, and chocolate on the day of urine collection. They emptied bladder before bed and collect all the urine outputs from 22:00 till the morning void on the next day. This sampling timeframe aims to collect the nocturnal secretion of melatonin under dim light while minimizing disruption to nocturnal sleep. Urine aMT6s were assayed by an ultra-performance liquid chromatography-negative electrospray ionization tandem mass spectrometry (UPLC-MS/MS) method (Waters Acquity Xevo TQD system, Waters Corporation, Milford, MA, USA)^8^. This method had a lower limit of quantitation at 0.5 nmol/L, with a linear analytical range up to 2500 nmol/L. The intra-assay coefficients of variation (CV) and inter-assay CV were 3.0% to 4.3%, and 4.2% to 5.1%, respectively ^9^. All aMT6s levels were creatinine-adjusted. Urine creatinine was measured by the kinetic Jaffé method, with a Cobas 8000 Modular Analyzer and manufacturer’s optimized reagents (F. Hoffmann-La Roche Ltd, Basel, Switzerland). Both the intra- and inter-assay CV for creatinine were < 3%.

## Supplement 3. Pupillometric measurements

All participants were interviewed by an experienced clinician for a detailed medical and psychiatric history and current usage of medications. Medical or eye conditions were identified through self-report and verified using the Clinical Management System, a centralized computer record covering over 90% of healthcare services with detailed diagnostic and treatment data in Hong Kong. Bedside pupil swing tests, visual acuity test, and the Ishihara color test were conducted to rule out presence of pupillary defects, blindness, and color blindness. All PIPR assessments were conducted in between 10 AM and 5 PM to minimize circadian variations^10^. The light protocol in this study was based on our previous work measuring PIPR in older individuals^11^. Pupils were not pharmacologically dilated. After 20-minutes of dark adaptation, a blue light stimulus (2.0 log lux, 463nm, FWHM 20nm) was first presented to both eyes for 1second(s) with post-stimulus continuous pupil size recording for 60s. The test is repeated with a red-light stimulus (2.0 log lux, 622nm, FWHM 20nm) for 1s with 2 minutes of rest in darkness in between the tests. Baseline pupil diameter (BPD) is measured as the average of 10s prior to the onset of each light stimulus. PIPR-6s was defined as the as the pupil size at 6s post-stimulus divided by the BPD to adjust for inter-individual pupil size at the start of the stimulus^12^. The PIPR-red was included as red light activates ipRGCs via cones (less so by rods) without direct activation of melanopsin, and serves as a control to quantify non-specific factors among the individuals^12^. The Net PIPR6s were reported by subtracting the PIPR-red from the PIPR-blue and was the pre-defined outcomes in this study.

## Supplement 4. Statistical analysis

The power estimation is based on a study of PIPR in PD patients^13^ which reported an effect size (Cohens’ d) of 0.8. We employed a more conservative effect size of 0.6, the estimated sample size would be 45 in each group to allow 80% power and an alpha of 0.05. The pupillometry data were cleaned for blinking and artefacts. The data were analyzed by customed-scripts in Python. The “cosinor” and “nparACT” packages in R were used to perform the cosinor and nonparametric analyses, respectively^14,15^. The baseline clinical characteristics, sleep measures, and PIPR metrics of the HC, iRBD and PD groups were compared by Chi’s Square tests and one-way ANOVA, where appropriate. The number of subjects with valid data was high (>95%), missing data were not imputed, the number of valid data were reported in the tables. Ordinal logistic regression models were used to assess the associations between PIPR, to the dependent ordinal variable which was arranged in ascending order of severity (i.e. HC < iRBD < PD). We include the time interval between PIPR examination time from actigraphic sleep midpoint and the maximal amplitude of constriction (MAC) to blue light as co-variates to control for the effect of circadian time of the PIPR and the inter-individual difference in pre-receptoral filtering, pupil diameter during the light stimulus, and retinal irradiance, respectively^11^. Generalized linear models (GLMs) were applied to examine the relationships between PIPR and the dependent variables of actigraphic amplitude, mesor, and acrophase, and urinary aMT6s. Linear regression analysis was conducted to examine the relationship between PIPR and the cognitive scores (HK-MoCA), controlling for confounders. Two-tailed p-value <0.05 was considered statistically significant. For multiple comparisons of the actigraphic rest-activity parameters, the p-values were further adjusted using the False Discovery Rate (FDR) method^16^. Sensitivity analyses conducted after excluding 3 iRBD participants taking antidepressants, and additional analysis excluding 18 participants using melatonin were conducted for sleep and circadian outcomes. Statistical tests were performed using SPSS (version 27.0) for Windows (IBM Corp, Armonk, NY) and R Studio (Version 4.2.3).

## eTable 1: Actigraphic rest-activity rhythms and melatonin

|  | All | HC^1^ | iRBD^2^ | PD^3^ | p-value | adj p-value^#^ | post-hoc test |
| --- | --- | --- | --- | --- | --- | --- | --- |
| Sleep-wake Pattern | | | | | | | |
|  | N=130 | N=44 | N=44 | N=42 |  |  |  |
| Rest onset | 23:38±01:11 | 23:49±01:10 | 23:39±01:08 | 23:25±01:13 | 0.30 | 0.43 |  |
| Sleep onset | 23:52±01:11 | 00:05±01:10 | 23:51±01:12 | 23:39±01:11 | 0.27 | 0.43 |  |
| Sleep offset | 07:13±01:22 | 07:22±01:13 | 07:22±01:29 | 06:54±01:22 | 0.20 | 0.40 |  |
| Rest offset | 07:31±01:20 | 07:25±01:16 | 07:48±01:26 | 07:18±01:15 | 0.18 | 0.40 |  |
| Sleep midpoint | 03:41±01:07 | 03:45±01:06 | 03:49±01:12 | 03:29±01:03 | 0.34 | 0.43 |  |
| TIB | 07:52±01:08 | 07:36±01:02 | 08:09±01:03 | 07:53±01:15 | 0.07 | 0.23 |  |
| Sleep latency | 00:14±00:13 | 00:16±00:15 | 00:13±00:10 | 00:14±00:14 | 0.54 | 0.54 |  |
| Total sleep time | 07:20±01:09 | 07:17±01:11 | 07:30±01:00 | 07:14±01:16 | 0.54 | 0.54 |  |
| WASO | 01:07±00:56 | 00:46±00:20 | 01:05±00:48 | 01:30±01:19 | **0.001** | **0.01** | 1<3 |
| Sleep efficiency | 78±12% | 83±7% | 78±12% | 74±15% | **0.003** | **0.02** | 1>3 |
| Circadian Rest-activity Rhythms | | | | | | | |
|  | N=132 | N=44 | N=45 | N=43 |  |  |  |
| IS | 0.57±0.13 | 0.60±0.13 | 0.59±0.13 | 0.52±0.14 | **0.008** | **0.01** | 1>3,2>3 |
| IV | 0.92±0.24 | 0.86±0.23 | 0.86±0.21 | 1.06±0.24 | **<0.001** | **0.003** | 1<3, 2<3 |
| RA | 0.86±0.10 | 0.87±0.12 | 0.89±0.05 | 0.82±0.12 | **0.006** | **0.01** | 2>3 |
| L5 (counts) | 13.2±9.8 | 13.6±10.7 | 12.4±6.0 | 13.5±12.0 | 0.82 | 0.82 |  |
| M10 (counts) | 186.5±84.8 | 209.4±84.3 | 211.6±73.0 | 136.8±76.5 | **<0.001** | **0.003** | 1>3, 2>3 |
| Mesor (counts) | 117.7±53.5 | 134.5±51.4 | 130.4±46.6 | 85.7±49.5 | **<0.001** | **0.003** | 1>3, 2>3 |
| Amplitude (counts) | 90.8±45.0 | 102.7±46.7 | 103.4±37.1 | 65.3±40.8 | **<0.001** | **0.003** | 1>3, 2>3 |
| Acrophase | 14.5±1.9 | 14.8±2.4 | 14.5±1.6 | 14.0±1.5 | 0.12 | 0.13 |  |
| Overnight urinary 6-sulfatoxymelatonin (aMT6s) ^^^ | | | | | | | |
|  | N=114 | N=45 | N=28 | N=41 |  |  |  |
| aMT6s adjusted | 16.8 ± 12.1 | 17.8±10.6 | 17.2±19.1 | 15.4±6.7 |  | 0.65 |  |

^#^ For multiple comparisons of the actigraphic rest-activity, the p-values within ‘sleep-wake pattern’ and ‘circadian RAR’, respectively, were further adjusted using the False Discovery Rate (FDR) method

^^^ Seventeen iRBD patients and one PD patient were on exogenous melatonin and were excluded from the urinary aMT6s collection. Additionally, three PD patients were unable to collect overnight urine samples due to impaired mobility.

Abbreviations: TIB: Time in bed, IS: Inter-daily stability, IV: Intra-daily variability, RA: Relative amplitude, MESOR: Midline Estimating Statistic of Rhythm, L5 = the average activity during the least active 5-hour period; M10 = the average activity during the most active 10-hour period

## eTable 2. Sensitivity analysis excluding subjects on exogenous melatonin

|  | All | HC^1^ | iRBD^2^ | PD^3^ | p-value | adj p-value | post-hoc test |
| --- | --- | --- | --- | --- | --- | --- | --- |
| Sleep-wake pattern | | | | | | | |
|  | N=112 | N=44 | N=27 | N=41 |  |  |  |
| Rest onset | 23:34±01:08 | 23:49±01:10 | 23:29±00:56 | 23:22±01:12 | 0.18 | 0.30 |  |
| Sleep onset | 23:49±01:08 | 00:05±01:10 | 23:42±00:59 | 23:37±01:11 | 0.15 | 0.30 |  |
| Sleep offset | 07:09±01:18 | 07:22±01:13 | 07:15±01:21 | 06:50±01:19 | 0.16 | 0.30 |  |
| Rest offset | 07:24±01:15 | 07:25±01:16 | 07:38±01:17 | 07:15±01:12 | 0.46 | 0.58 |  |
| Sleep midpoint | 03:37±01:02 | 03:45±01:06 | 03:40±00:58 | 03:26±01:01 | 0.37 | 0.53 |  |
| TIB | 07:49±01:10 | 07:36±01:02 | 08:09±01:11 | 07:52±01:16 | 0.15 | 0.30 |  |
| Sleep latency | 00:15±00:14 | 00:16±00:15 | 00:13±00:11 | 00:15±00:14 | 0.76 | 0.76 |  |
| Total sleep time | 07:19±01:12 | 07:17±01:11 | 07:32±01:07 | 07:13±01:16 | 0.55 | 0.61 |  |
| WASO | 01:07±00:56 | 00:46±00:20 | 01:11±00:59 | 01:28±01:18 | **0.005** | **0.025** | 1<3 |
| Sleep efficiency | 80±19% | 87±23% | 78±15% | 74±15% | **0.005** | **0.025** | 1>3 |
| Circadian rest-activity Rhythms | | | | | | | |
|  | N=114 | N=44 | N=28 | N=42 |  |  |  |
| IS | 0.57±0.13 | 0.60±0.13 | 0.60±0.13 | 0.53±0.12 | **0.02** | **0.03** | 1>3 |
| IV | 0.93±0.25 | 0.86±0.23 | 0.85±0.21 | 1.06±0.25 | **<0.001** | **0.003** | 1<3, 2<3 |
| RA | 0.86±0.11 | 0.87±0.12 | 0.90±0.05 | 0.82±0.12 | **0.008** | **0.02** | 2>3 |
| L5 | 13.1±10.2 | 13.6±10.7 | 11.5±5.6 | 13.7±12.1 | 0.62 | 0.62 |  |
| M10 | 186.2±86.6 | 209.4±84.3 | 220.7±75.4 | 139.0±76.1 | **<0.001** | **0.003** | 1>3, 2>3 |
| Mesor | 117.2±54.4 | 134.5±51.4 | 130.1±48.0 | 87.1±49.2 | **<0.001** | **0.003** | 1>3, 2>3 |
| Amplitude | 90.5±46.1 | 102.7±46.7 | 107.9±37.9 | 66.2±40.8 | **<0.001** | **0.003** | 1>3, 2>3 |
| Acrophase | 14.4±1.9 | 14.8±2.3 | 14.5±1.6 | 14.0±1.5 | 0.11 | 0.12 |  |

## eTable 3. Pupillometric measurements

|  | All | HC^1^ | iRBD^2^ | PD^3^ | p-value | Post-hoc test |
| --- | --- | --- | --- | --- | --- | --- |
|  | N=135 | N=45 | N=45 | N=45 |  |  |
| Full sample analysis | mean ± SD | | | |  |  |
| Uncorrected visual acuity (logMAR) | 0.51±0.26 | 0.45±0.25 | 0.54±0.26 | 0.55±0.27 | 0.14 |  |
| Clock time of PIPR exam | 13:04±02:09 | 12:47±02:03 | 13:11±02:17 | 13:15±02:09 | 0.54 |  |
| Time of PIPR exam since midsleep | 09:21±02:21 | 09:05±02:17 | 09:20±02:28 | 09:39±02:18 | 0.53 |  |
| Daylength ^a^ | 12:31±00:45 | 12:31±00:32 | 12:29±00:53 | 12:32±00:49 | 0.97 |  |
|  |  |  |  |  |  |  |
| BPD_Blue (mm) | 5.7 ± 0.8 | 5.7 ± 0.8 | 5.6 ± 0.8 | 5.7 ± 0.8 | 0.74 |  |
| PIPR6s_Blue ^b^ | 28.4 ± 12.0 | 34.0 ± 10.6 | 28.5 ± 11.7 | 22.8 ± 11.4 | **<0.001** | 1>3 |
| BPD_Red (mm) | 5.6 ± 0.8 | 5.6 ± 0.8 | 5.6 ± 0.8 | 5.6 ± 0.8 | 0.90 |  |
| PIPR6s_Red ^b^ | 9.9 ± 4.6 | 10.2 ± 5.0 | 9.9 ± 4.1 | 9.5 ± 4.9 | 0.79 |  |
|  | | | | | | |
| Full sample analysis | | | | | | |
|  | N=135 | N=45 | N=45 | N=45 |  |  |
| Net PIPR-6s ^b^ | 18.6 ± 10.8 | 23.8 ± 9.4 | 18.6 ± 10.8 | 13.3 ± 9.6 | **<0.001** | 1>2>3 |
|  |  |  |  |  |  |  |
| Sensitivity analysis excluding iRBD patients taking an antidepressant | | | | | | |
|  | N=135 | N=45 | N=42 | N=45 |  |  |
| Net PIPR-6s ^b^ | 18.8 ± 10.7 | 23.8±9.4 | 19.2 ±10.5 | 13.3±10.7 | **<0.001** | 1>2>3 |

Abbreviations: SD: Standard deviation; PIPR: Post-illumination pupillary response; PVA: Presenting visual acuity (uncorrected visual acuity); BPD: Baseline pupil diameter; MAC: Maximum constriction amplitude.

^a^ The daylength on the day of PIPR examination was obtained from the Hong Kong Observatory.

^b^ All PIPR metrics are presented as a percentage of the baseline pupil diameter

## eTable 4. Ordinal logistic regression analysis

|  | PseudoR^2^  (Nagelkerke) | Estimate | Odd Ratio | 95% Confidence interval | | p-value |
| --- | --- | --- | --- | --- | --- | --- |
| Net PIPR-6s | | | | | | |
| Full sample analysis | | | | | | |
| Model 1 | 0.22 | -0.075 | 0.93 | 0.89 | 0.96 | **<0.001** |
| Model 2 | 0.35 | -0.086 | 0.92 | 0.88 | 0.95 | **<0.001** |
|  | | | | | | |
| Sensitivity analysis excluding iRBD patients taking an antidepressant | | | | | | |
| Model 1 | 0.23 | -0.079 | 0.92 | 0.89 | 0.96 | **<0.001** |
| Model 2 | 0.36 | -0.091 | 0.91 | 0.87 | 0.95 | **<0.001** |

Ordinal variables: the categories were arranged in ascending order of severity (i.e., controls < iRBD < PD).

Dependent variables: Net PIPR6s in percentage, normalized to baseline pupil size

Odd ratio is obtained by exponentiate the estimate

Model 1: adjusted for age and gender

Model 2: adjusted for age, gender, visual acuity, daylength on the day of PIPR exam, MAC to blue light stimulus, PIPR examination time since actigraphic sleep midpoint, PHQ-9 and ISI scores

## eTable 5. Generalized linear model of Net PIPR on circadian parameters

|  | AIC | B | 95% Confidence interval | p-value |
| --- | --- | --- | --- | --- |
| **Amplitude** | | | | |
| Full sample analysis | | | | |
| Model 1 | 1363.3 | 0.010 | 0.003, 0.017 | 0.007 |
| Model 2 | 1334.0 | 0.016 | 0.006, 0.025 | 0.001 |
|  | | | | |
| Sensitivity analysis among participants not taking melatonin | | | | |
| Model 1 | 1123.1 | 0.013 | 0.006, 0.021 | 0.001 |
| Model 2 | 1109.4 | 0.021 | 0.011, 0.031 | <0.001 |
|  | | | | |
| Sensitivity analysis excluding iRBD patients taking an antidepressant | | | | |
| Model 1 | 1329.6 | 0.012 | 0.004, 0.019 | 0.002 |
| Model 2 | 1299.7 | 0.018 | 0.008, 0.027 | <0.001 |
|  | | | | |
| **Mesor** | | | | |
| Full sample analysis | | | | |
| Model 1 | 1408.5 | 0.012 | 0.005, 0.018 | 0.001 |
| Model 2 | 1379.1 | 0.016 | 0.007, 0.024 | <0.001 |
|  | | | | |
| Sensitivity analysis among participants not taking melatonin | | | | |
| Model 1 | 1154.5 | 0.015 | 0.008, 0.022 | <0.001 |
| Model 2 | 1140.5 | 0.021 | 0.012, 0.030 | <0.001 |
|  | | | | |
| Sensitivity analysis excluding iRBD patients taking an antidepressant | | | | |
| Model 1 | 1374.9 | 0.013 | 0.006, 0.020 | <0.001 |
| Model 2 | 1345.0 | 0.017 | 0.009, 0.026 | <0.001 |
|  | | | | |
| **Acrophase** | | | | |
| Full sample analysis | | | | |
| Model 1 | 573.2 | 0.001 | -0.001, 0.003 | 0.29 |
| Model 2 | 535.2 | 0.001 | -0.001, 0.004 | 0.34 |
|  | | | | |
| Full sample analysis | | | | |
| **Overnight urinary aMT6s** | | | | |
| Model 1 | 834.5 | -0.002 | -0.011, 0.008 | 0.72 |
| Model 2 | 818.5 | -0.002 | -0.015, 0.011 | 0.79 |

Abbreviations: AIC: Akaike’s Information Criterion

Model 1 adjusted for age and gender.

Model 2 additionally adjusted for day length, MAC to blue light stimulus, PIPR time interval from actigraphic sleep midpoint, acrophase of actigraphic light exposure.

## eTable 6. Linear regression analysis of Net PIPRs with HK-MoCA score

|  | B | Standardized Coefficients  Beta | 95% Confidence interval | p-value |
| --- | --- | --- | --- | --- |
| Full sample analysis | | | | |
| Unadjusted model | 0.052 | 0.220 | 0.012, 0.092 | 0.01 |
| Adjusted model | 0.046 | 0.193 | 0.007, 0.085 | 0.02 |
|  |  |  |  |  |
| Sensitivity analysis excluding iRBD patients taking an antidepressant | | | | |
| Unadjusted model | 0.045 | 0.190 | 0.004, 0.085 | 0.03 |
| Adjusted model | 0.038 | 0.163 | 0.000, 0.077 | 0.05 |

Model adjusted for age, gender, education, ISI and PHQ-9 scores

Abbreviation: ISI: Insomnia Severity Index; PHQ-9: Patient Health Questionnaire-9, PIPR: post-illumination pupillary response

## Reference

1.	Hughes AJ, Daniel SE, Kilford L, Lees AJ. Accuracy of clinical diagnosis of idiopathic Parkinson's disease: a clinico-pathological study of 100 cases. *Journal of neurology, neurosurgery, and psychiatry*. Mar 1992;55(3):181-4. doi:10.1136/jnnp.55.3.181

2.	Medicine AAoS. *International Classification of Sleep Disorders. 3rd ed.* American Academy of Sleep Medicine; 2014.

3.	Yeung PY, Wong LL, Chan CC, Leung JL, Yung CY. A validation study of the Hong Kong version of Montreal Cognitive Assessment (HK-MoCA) in Chinese older adults in Hong Kong. *Hong Kong Med J*. Dec 2014;20(6):504-10. doi:10.12809/hkmj144219

4.	Stoschitzky K, Sakotnik A, Lercher P, et al. Influence of beta-blockers on melatonin release. Clinical Trial

Randomized Controlled Trial

Research Support, Non-U.S. Gov't. *Eur J Clin Pharmacol*. Apr 1999;55(2):111-5.

5.	Murphy PJ, Myers BL, Badia P. Nonsteroidal anti-inflammatory drugs alter body temperature and suppress melatonin in humans. *Physiol Behav*. Jan 1996;59(1):133-9. doi:10.1016/0031-9384(95)02036-5

6.	Bourne RS, Mills GH. Melatonin: possible implications for the postoperative and critically ill patient. *Intensive Care Med*. Mar 2006;32(3):371-9. doi:10.1007/s00134-005-0061-x

7.	Graham C, Cook MR, Kavet R, Sastre A, Smith DK. Prediction of nocturnal plasma melatonin from morning urinary measures. *J Pineal Res*. May 1998;24(4):230-8. doi:10.1111/j.1600-079x.1998.tb00538.x

8.	Li W, Kwok CC, Chan DC, et al. Disruption of sleep, sleep-wake activity rhythm, and nocturnal melatonin production in breast cancer patients undergoing adjuvant chemotherapy: prospective cohort study. *Sleep Med*. Mar 2019;55:14-21. doi:10.1016/j.sleep.2018.11.022

9.	Feng H, Ho AW, Lei B, et al. Dim light melatonin patterns in unaffected offspring of parents with bipolar disorder: A case-control high-risk study. *J Affect Disord*. Oct 15 2022;315:42-47. doi:10.1016/j.jad.2022.07.029

10.	Zele AJ, Feigl B, Smith SS, Markwell EL. The circadian response of intrinsically photosensitive retinal ganglion cells. *PLoS One*. Mar 14 2011;6(3):e17860. doi:10.1371/journal.pone.0017860

11.	Chan JWY, Li CT, Chau SWH, et al. Attenuated melanopsin-mediated post-illumination pupillary response is associated with reduced actigraphic amplitude and mesor in older adults. *Sleep*. Feb 10 2025;48(2)doi:10.1093/sleep/zsae239

12.	Kelbsch C, Strasser T, Chen Y, et al. Standards in Pupillography. *Front Neurol*. 2019;10:129. doi:10.3389/fneur.2019.00129

13.	Joyce DS, Feigl B, Kerr G, Roeder L, Zele AJ. Melanopsin-mediated pupil function is impaired in Parkinson's disease. *Sci Rep*. May 17 2018;8(1):7796. doi:10.1038/s41598-018-26078-0

14.	Sachs M. Cosinor: Tools for Estimating and Predicting the Cosinor Model. . R Package Version 1.2.3 , 2023. Accessed 1.11.2024, <https://cran.r-project.org/web/packages/cosinor/index.html>

15.	Blume C, Santhi, N., Schabus, M. 'nparACT' package for R: a free software tool for the non-parametric analysis of actigraphy data. . *MethodsX*. 2016;(3):430-435.

16.	Benjamini Y, Hochberg Y. Controlling the false discovery rate - a practical and powerful approach to multiple testing. *J R Stat Soc B*. 1995;57:289-230.
